# The design of an evaluation framework for diabetes self-management education and support programs delivered nationally

**DOI:** 10.1186/s12913-021-07374-4

**Published:** 2022-01-09

**Authors:** Jenny Louise Olson, Becky White, Helen Mitchell, Jennifer Halliday, Timothy Skinner, Deborah Schofield, Jennifer Sweeting, Natasha Watson

**Affiliations:** 1 Diabetes WA, Level 3, 322 Hay Street, Subiaco, Western Australia; 2 Department of Kinesiology, The Pennsylvania State University, 276 Recreation Hall, University Park, State College, PA 16802 USA; 3 College of Medicine, The Pennsylvania State University, Hershey, PA USA; 4 School of Psychology, Deakin University, Geelong, Victoria Australia; 5 The Australian Centre for Behavioural Research in Diabetes, Diabetes Victoria, Melbourne, Victoria Australia; 6 La Trobe Rural Health School, La Trobe University, Bendigo, Victoria Australia; 7 Institute of Psychology, University of Copenhagen, Copenhagen, Denmark

**Keywords:** Implementation science, Program evaluation, Diabetes self-management education and support, Health services research, Self-management, Chronic disease, Diabetes mellitus, Self-determination, Psychosocial adjustment

## Abstract

**Background:**

The aim of this work was to develop a National Evaluation Framework to facilitate the standardization of delivery, quality, reporting, and evaluation of diabetes education and support programs delivered throughout Australia through the National Diabetes Services Scheme (NDSS). The NDSS is funded by the Australian Government, and provides access to diabetes information, education, support, and subsidized product across diverse settings in each state and territory of Australia through seven independent service-providers. This article reports the approach undertaken to develop the Framework.

**Methods:**

A participatory approach was undertaken, focused on adopting nationally consistent outcomes and indicators, nominating objectives and measurement tools, specifying evaluation processes, and developing quality standards. Existing programs were classified based on related, overarching indicators enabling the adoption of a tiered system of evaluation.

**Results:**

Two outcomes (i.e., improved clinical, reduced cost) and four indicators (i.e., improved knowledge and understanding, self-management, self-determination, psychosocial adjustment) were adopted from the Eigenmann and Colagiuri national consensus position statement for diabetes education. This allowed for the identification of objectives (i.e., improved empowerment, reduced distress, autonomy supportive program delivery, consumer satisfaction) and related measurement instruments. Programs were categorized as comprehensive, topic-specific, or basic education, with comprehensive programs allocated to receive the highest-level of evaluation. Eight quality standards were developed, with existing programs tested against those standards. Based on the results of testing, two comprehensive (OzDAFNE for people with type 1 diabetes, DESMOND for people with type 2 diabetes), and eight topic-specific (CarbSmart, ShopSmart, MonitorSmart, FootSmart, MedSmart, Living with Insulin, Insulin Pump Workshop, Ready Set Go – Let’s Move) structured diabetes self-management education and support programs were nominated for national delivery.

**Conclusions:**

The National Evaluation Framework has facilitated consistency of program quality, delivery, and evaluation of programs delivered by multiple service providers across diverse contexts. The Framework could be applied by other service providers who facilitate multiple diabetes education and support programs and could be adapted for use in other chronic disease populations where education and support are indicated.

**Supplementary Information:**

The online version contains supplementary material available at 10.1186/s12913-021-07374-4.

## Background

Diabetes is a chronic condition characterized by elevated blood glucose levels and is associated with increased risk of serious health complications [1]. It is estimated that more than 451-million adults are living with diabetes globally, with projections indicating this could increase to 693 million by 2024 [2]. Approximately 5-million deaths were attributed to the condition globally during 2017 [2]. Diabetes also results in significant financial burden, with related global healthcare expenditure estimated at USD$850 billion annually [2]. Diabetes self-management education and support programs (DSMES) provided by consumer health organizations and health professionals have the potential to significantly improve a person’s ability to live well with diabetes [3] and reduce risk of diabetes-related complications and health system burden [4]. Given the rising prevalence of diabetes and the large financial burden attributed to the condition, the need to evaluate the outcomes of programs implemented in community settings has never been more paramount.

The Australian Government invests significantly in diabetes-related services and care. Since 1987, programs, services, and access to diabetes product has been facilitated through the National Diabetes Services Scheme (NDSS), an initiative of the Australian Government, administered by Diabetes Australia [5]. The aim of the NDSS is to enhance the capacity of people with diabetes and to live a life in which the impact of diabetes is minimized. Seven state and territory Agents (herein referred to as ‘Agents’) receive funding through the NDSS to support people living with diabetes, across diverse settings. As with many national programs for health service delivery, the types of programs and services offered, the objectives targeted within programs, and the evaluation processes employed by individual Agents varied extensively. This heterogeneity across organizations and programs impeded consistent evaluation of the reach, outcomes, and impact of the NDSS.

Some programs offered through the NDSS are backed by extensive evidence garnered from research studies. These included Dose Adjustment or Normal Eating (DAFNE; with the Australian edition entitled ‘OzDAFNE’) for people with type 1 diabetes [6–10]; and Diabetes Education and Self-Management for Ongoing and Newly Diagnosed (DESMOND) for people with type 2 diabetes [11–14]. However, evidence of the effectiveness of many other programs offered through the NDSS was limited, as was evaluation of the outcomes of DESMOND and OzDAFNE delivered across service providers and diverse contexts nationally. Without direct and consistent national evaluation of program outcomes, the broader real-world impact of NDSS programs was unclear. Given the substantive expenditure of public funds, it is important to demonstrate program outcomes and impact, and continually assess and improve the quality of services provided. Thus, the need for a standardized national approach to evaluation of programs facilitated through the NDSS was identified. A recent policy shift by the Australian Government to outcome-based models and funding further reinforced the need for systematic evaluation of NDSS programs.

Internationally, existing frameworks have been adapted for the purpose of evaluating diabetes programs. In the United States for example, the Centers for Disease Control and Prevention’s (CDC) Prevention’s Framework for Program Evaluation in Public Health has guided the evaluation of outcomes of the National Diabetes Education Program [15, 16]. Similarly, the Reach, Effectiveness, Adoption, Implementation, and Maintenance (RE-AIM) framework guided the evaluation of the National Diabetes Prevention Program [17, 18]. In Canada, The Diabetes Evaluation Framework (DEFINE) was developed as a flexible tool for the evaluation of diabetes care, programs, and services, that could be applied by different users and in different settings [19]. These programs provide exemplars of the successful application of generic evaluation frameworks to evaluate individual programs delivered at a national level (i.e., the programs delivered in the United States), and a broad framework for evaluating heterogeneous types of health care, services, and programs. In the Australian setting however, in addition to the need for standardized evaluation processes and reporting, there was also a need to synthesize the suite of NDSS programs offered by independent service providers across varied settings, and relatedly, for the development of overarching quality standards.

Therefore, the aim of this work was to systematically develop a National Evaluation Framework to: (a) facilitate national standardization of group-based diabetes programs and evaluation throughout Australia, (b) ensure consistent program quality and reporting by NDSS service providers, and (c) facilitate consistent evaluation processes and reporting of overall program outcomes. To our knowledge, this is the first framework for diabetes self-management education that describes the process of standardization of DSMES and evaluation processes at a national level.

## Methods

### Design

**Fig. 1:**
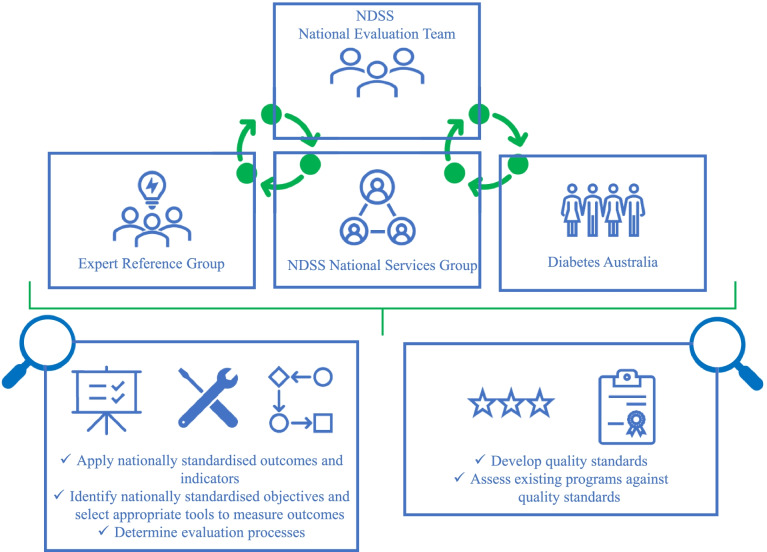
Approach to develop the National Evaluation Framework

A participatory approach was adopted to achieve the aims of the research, with two key areas of focus, including the development of: (1) nationally standardized outcomes and indicators; program categories; objectives and measurement tools; and evaluation processes; and (2) quality standards and assessment procedures. The approach and areas of focus are depicted in Fig. 1 and described in greater detail below.

### Participatory approach

A National Evaluation Team (NET) was established to lead the development and ongoing implementation of the National Evaluation Framework. The team was comprised of research and evaluation experts employed by Diabetes WA, with funding support from the NDSS. The Team met regularly with representatives from Diabetes Australia, who provided project governance and oversight.

An Expert Reference Group (ERG) was established to guide the design, development, and implementation of the Framework. The group included representatives from Diabetes Australia, the Australian Centre for Behavioural Research in Diabetes, The Australian Diabetes Educators Association﻿, Deakin University, Charles Darwin University, a consumer representative, and Agents.

The NET and ERG consulted widely with the NDSS National Services Group, a diverse group of health professionals working with people with diabetes (e.g., Dietitians, Diabetes Educators, and Health Service Managers) representing Agents. The range of health professionals providing diabetes services in Australia is diverse, both organizationally and geographically. Therefore, understanding the viewpoints of a broad range of experts was integral in shaping the design and implementation of the Framework.

### Nationally standardized outcomes and indicators, program categories, objectives and measurement tools, and evaluation processes

Research evidence guided the identification of outcomes, indicators, and objectives for diabetes programs and services that were most likely to lead to favorable outcomes. To support nationally consistent program delivery and evaluation and ensure the best opportunity to improve the outcomes of people with diabetes, outcomes and indicators were adopted from the national consensus position statement previously developed on behalf of Diabetes Australia [20]. Informed by an international evidence-base and extensive consultation, the position statement outlines three key goals for diabetes education, including: (1) optimal adjustment to living with diabetes, (2) optimal health outcomes, and (3) optimal cost effectiveness. Goals 2 and 3 recognize the potential impact of diabetes education on physical health outcomes and optimal cost effectiveness. However, the statement acknowledges that it is challenging to directly attribute these to diabetes education, and thus, attention should primarily focus on Goal 1. Components or ‘indicators’ of diabetes education identified as relating to the goal of optimal adjustment to living with diabetes include: (a) knowledge and understanding, (b) self-management (i.e., diabetes self-care skills and behaviors), (c) self-determination and (d) psychological adjustment.

The identification of outcomes and indicators for DSMES allowed for the categorization of existing diabetes programs based on the indicators targeted within those programs. This required the identification of group-based programs currently delivered throughout Australia, and the collection of information related to those programs. In order to ascertain what programs were being delivered nationally, Agents from each state and territory logged information about the programs they offered, including the population targeted (e.g., people with type 1 diabetes), targeted outcomes, and duration of each program, in a national register held by Diabetes Australia. Members of the NET, in collaboration with state and territory Agent representatives, the National Services Group, and Diabetes Australia then compared the outcomes of each program included in the register with those specified in the National Evaluation Framework (i.e., each program was assessed as to whether it targeted knowledge and understanding, self-management, self-determination, and/or psychological adjustment). In instances where it was not clear whether a program outcome matched one or more of the outcomes specified in the Framework, further clarification was sought from Agent representatives to better understand the nature of that program. Together, the group categorized the programs that had been presented by state and territory Agents of the NDSS according to the number and types of outcomes targeted in the National Evaluation Framework.

The categorization of programs enabled the specification of a three-tiered system of evaluation, allowing for differential program evaluation, dependent on the nature of the programs ascribed to each tier. More comprehensive evaluation processes were ascribed to resource-intensive, higher-cost programs predicted to demonstrate the greatest behavioral impact (Tier 3). Less intensive evaluation processes were ascribed to moderately resource intensive programs predicted to demonstrate a more modest degree of behavioral impact (Tier 2); and the least intensive evaluation processes were ascribed to the lowest cost, most basic programs (Tier 1). Program objectives were then defined for each evaluation tier, and appropriate measurement tools selected to assess outcomes against those objectives. Potential participant burden was weighed against the benefits of intensive program evaluation. Moreover, pragmatic assessment of program objectives had to be applied across the broad range of programs being delivered.

The selection of measurement instruments was guided by a recent review of psychometric tools for diabetes education services [21]. The review investigated instruments to measure commonly targeted outcomes of diabetes education. Instruments were assessed on suitability, validity, reliability, feasibility, and sensitivity. Just three of the 37 tools evaluated met all five criteria. Several other instruments were deemed suitable as they met all but one of the criteria. Based on these findings, potential instruments to measure targeted objectives were selected for consideration by the ERG. Specific instruments were then nominated for inclusion in the National Evaluation Framework. Estimated time required to complete measures ranges from approximately 5-10 min for the most basic level of assessment, to around 20 min for the most complex level of assessment. A combination of online and paper-based methods of dissemination were utilized. States and territories used standardized spreadsheet to collate data for each program before sending it to the NET. Assessments at baseline and post-participation were collected by program educators. For follow-up assessments, measures were mailed to participants and returned in postage-paid envelopes.

### Quality standards and assessment

A set of quality standards was developed to elucidate processes of care and to ensure that NDSS programs were of high-quality and contained components to elicit key outcomes associated with optimal diabetes self-management. DSMES can have a significant positive effect on health [22]. For example, DSMES are effective in reducing blood glucose levels and improving psychosocial outcomes in people with type 2 diabetes [23–25] and type 1 diabetes [24]. Such programs have also been assessed as cost effective in empowering people with diabetes to self-manage their condition and mitigate risks of complications, with an incremental cost-effectiveness ratio of USD $5047 per additional quality-adjusted life year, when compared to usual care [26]. Therefore, it was important that the quality standards supported the delivery of programs that were structured and tailored to support the needs of the individual.

The content of the quality standards was informed by existing national and international standards and guidelines [27–30], state and federal government policy for primary care, chronic conditions, and diabetes [31, 32], and the National Safety and Quality Health Service Standards (NSQHS [33];). Collectively, these standards and guidelines recommend that DSMES should be person-centered (i.e., responsive to the unique needs of the individual), and provided at the time of diagnosis and throughout the person’s journey with diabetes. It is also recommended that DSMES should be accessible, culturally appropriate, and provide appropriate information and education for all people with diabetes, their families, and carers. The importance of strategies promoting active learning, goal setting, and supported decision making is also recognized. Programs should have a written curriculum, standardized facilitator training, and a quality development pathway to ensure fidelity and facilitate quality assurance.

Existing topic specific and comprehensive NDSS DSMES were assessed in accordance with the newly developed standards, to ensure consistent quality across all states and territories. In September 2016, representatives of each Agent assessed their existing DSMES utilizing a user-friendly self-assessment tool, developed to guide the application of the quality standards into practice. The tool is presented in Additional file 1. Agent representatives were engaged as Health Services Managers or Program Managers in their respective organizations. Agent representatives attended a one and a half day training workshop facilitated by the NET. Attendees were familiarized with the quality standards and self-assessment tools, and then had the opportunity to apply the tool to one of their organization’s programs, under the guidance of the workshop facilitators.

To mitigate risk of self-report bias, the evaluations of DSMES conducted by each of the Agents were independently reviewed by two members of the NET. The reviewers then met to discuss the outcomes of the independent assessments and whether individual programs met quality standards. Discrepancies between reviewers were identified and resolved through mutual agreement. Feedback was then provided to the NDSS Agents, including areas for quality improvement. Behavior change outcomes were not expected from tier 1 basic education programs; thus, no formal review of these programs was undertaken. However, the standards provide a general guide for the provision of basic education programs through the NDSS.

As the primary purpose of this work was to improve the quality of service delivered by an organization, the work was exempt from formal requirements for ethical approval and consent in accordance with the Australian Government’s National Health and Medical Research Council policy on ethical considerations in quality assurance and evaluation activities [34].

## Results

**Fig. 2:**
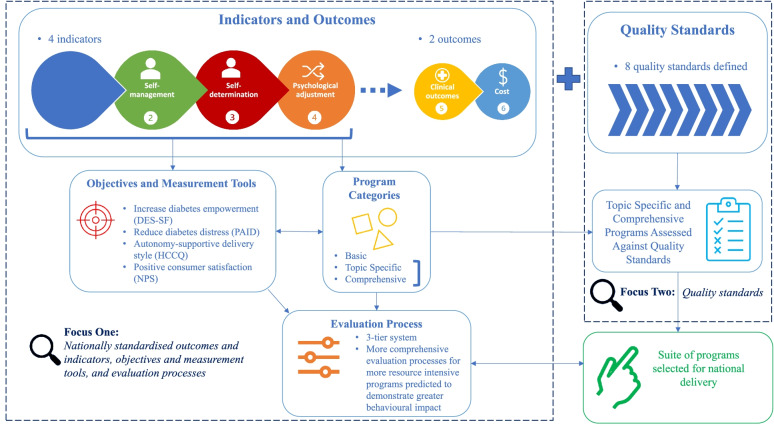
National Evaluation Framework

A National Evaluation Framework for diabetes education and support programs facilitated through the NDSS was developed. The Framework consists of nationally standardized outcomes and indicators, program categories, objectives, measurement tools, evaluation processes, and quality standards. These are summarized in Fig. 2.

### Outcomes and indicators, program categories, objectives and measurement tools, and evaluation processes

#### Outcomes and indicators

Four indicators (i.e., improved knowledge and understanding, self-management, self-determination, and psychological adjustment) and two outcomes (i.e., improved clinical and reduced cost) derived from the National Consensus Position Statement [20] were included in the NDSS National Evaluation Framework. These outcomes and indicators represent the aims of DSMES facilitated throughout Australia via the NDSS.

#### Program categories

Programs were classified into three distinct categories. Low intensity, less structured programs targeting knowledge and understanding and delivered to large groups, were classified as ‘basic education’. These programs were characterized as high-reach and low-cost and did not incorporate behavioral strategies to foster improvements in self-management, self-determination, or psychological adjustment. Thus, while basic education programs addressed the knowledge and understanding indicator, these programs were assessed as unlikely to address indicators of self-management, self-determination, or psychological adjustment.

More intensive, targeted DSMES focusing on a specific diabetes self-management topic (e.g., foot care) and addressing knowledge and understanding, in addition to at least one other Framework indicator (i.e., self-management, self-determination, or psychological adjustment) were classified as ‘topic specific’. Topic specific DSMES were short in duration (e.g., 3-h), delivered in smaller groups, and anticipated to be higher cost with lower reach than basic education programs.

Group DSMES that were of longer duration, provided structured self-management education covering a range of diabetes-related topics, and targeted all four Framework indictors (i.e., knowledge and understanding, self-management, self-determination, and psychological adjustment) were classified as ‘comprehensive’. Comprehensive DSMES are highly structured and resource intensive, with higher estimated cost per participant and lower reach than programs classified at other levels. However, such programs were anticipated to be the most effective in eliciting behavioral change.

#### Objectives and measurement tools

The burden of measuring constructs related to all four indicators was deemed too great and the assessment of constructs related to self-determination and psychological adjustment were prioritized. Moreover, direct evaluation of clinical outcomes and cost was not feasible, given the available resources, accessibility of information, and scope of the NDSS (e.g., Agents do not have access to medical records for evaluation of clinical outcomes). Likewise, intensive longitudinal surveillance necessary to determine cost savings would require a substantial financial investment beyond current levels of resourcing and access to information not currently available to Agents or evaluators.

Four objectives related to self-determination and psychological adjustment were identified for DSMES, including: (a) increased diabetes-related empowerment, (b) participant perceptions that facilitators were autonomy supportive, (c) reduced diabetes-related distress, and (d) positive consumer satisfaction. Four instruments were selected to measure outcomes against these objectives.

##### Increased diabetes-related empowerment

Diabetes-related empowerment refers to an individual’s perceived psychosocial self-efficacy to manage diabetes [35], and is positively associated with diabetes knowledge, medication adherence, and self-care behaviors [36]. The diabetes empowerment scale - short form (DES-SF) [35] was selected to measure this construct. The DES-SF provides a reliable 8-item measure of diabetes-related psychosocial self-efficacy (α = 0.84). Items are statements reflecting beliefs of empowerment and confidence to manage diabetes (e.g., “I can make choices about my diabetes management that are right for me.”). Responses are collected on 5-point scales (1 = *strongly disagree* and 5 = *strongly agree*). Higher scores indicate greater diabetes-related empowerment.

##### Perceptions of autonomy support

The health care climate questionnaire (HCCQ [37];) was selected to evaluate a construct related to the indicator of self-determination. Perceptions of autonomy support from health care providers have been associated with significant improvements in autonomous motivation for glucose control and reductions in HbA1c among people receiving treatment for diabetes [38]. Moreover, facilitator delivery style may influence the effectiveness of DSMES [34]. Inclusion of the HCCQ provided a mechanism of monitoring the fidelity of program delivery by educators specifically trained in a person-centered approach. Scores on the HCCQ demonstrate high internal reliability (α = 0.95) [37]. A modified 10-item version of the scale was applied, including the items most relevant to the person-centered delivery style associated with effective DSMES (α = 0.76). Items are statements reflecting perceived autonomy support (e.g., I feel that the staff has provided me with choices and options.” Responses are collected on 5-point scales (1 = *not true at all* and 5 = *very true*). Higher scores represent greater perceived autonomy support from health care providers.

##### Reduced diabetes-distress

Diabetes distress refers to psychosocial distress specifically related to the burden of living with, and managing, diabetes and its complications [39]. Diabetes distress is highly prevalent and associated with sub-optimal self-care and poorer emotional well-being [40]. The 20-item Problem Areas in Diabetes Scale (PAID; 41) was selected to measure diabetes distress. Items build on the stem “Which of the following diabetes issues are currently a problem for you?” (e.g., “Feeling discouraged with your diabetes treatment plan”). Responses are collected on a 5-point Likert scale (0 = *not a problem* and 4 = *serious problem*). Higher scores indicate greater diabetes-related distress. Scores on the scale demonstrate high internal reliability (α = 0.95) and concurrent validity has been established [41].

##### Consumer satisfaction

A global measure of consumer satisfaction was also adopted. Net Promotor Score (NPS [42, 43];) reflects the proportion of participants likely to recommend a program to others (i.e., promoters), minus the proportion of participants defined as detractors (i.e., those not likely to recommend the program). NPS consists of a single item, with responses collected on an 11-point scale (0 = *not at all likely to recommend to others* and 10 = *extremely likely to recommend to others*). The NPS score is calculated by subtracting the proportion of those who responded from 0-6 on the scale (‘detractors’) from the proportion who responded 9- 10 (‘promoters’). Higher NPS scores indicate greater participant satisfaction. NPS scores are compared against international health industry averages, as reflected in the International NPS & CX Benchmarks report [42].

#### Evaluation processes

Basic education programs were nominated at the first tier of evaluation. Evaluation focused on knowledge and understanding, and consumer satisfaction. As basic programs aim only to increase knowledge and understanding, with no focus on behavioral change, evaluation of tier one programs excluded evaluation of diabetes empowerment, distress, and autonomy support, and measures were administered immediately post-program.

DSMES classified as topic specific were ascribed to the second tier of evaluation. Evaluation included assessment of knowledge and understanding, confidence for self-management, self-determination, and psychological adjustment. As behavioral change was anticipated from DSMES within this tier, data collection was planned to take place pre- and post-program participation to assess changes in outcome measures.

DSMES classified as comprehensive were nominated to the third, and highest, evaluation tier. More complex evaluation was planned, with assessment of all behavior change objectives relating to diabetes empowerment and diabetes distress. Data collection was planned for three time points including pre- and post-program, and follow-up (e.g.at 3- or 12-months). The final NDSS programs included within each category, the indicators addressed within each of those categories and the ascribed evaluation tiers and evaluation processes relevant to each tier are presented in Table 1.

**Table 1:** NDSS program categories and evaluation tiers

Category	Indicators addressed	Programs	Tier	Evaluation process
Comprehensive DSMES	∙ Knowledge and understanding ∙ Self-management ∙ Self-determination ∙ Psychological adjustment	OzDAFNE (type 1 diabetes) DESMOND (type 2 diabetes)	3	∙ Measures of empowerment and reduced diabetes distress. ∙ Highest cost-per-head and lowest reach but most opportunity for behavioral impact. ∙ Complex evaluation, involving pre, post and 3-month follow up surveys.
Topic Specific DSMES	∙ Knowledge and understanding Plus, at least one of the following: ∙ Self-management ∙ Self-determination ∙ Psychological adjustment	CarbSmart ShopSmart MonitorSmart FootSmart MedSmart Living with Insulin Pump Workshop Ready Set Go, Let’s Move	2	∙ Measures of knowledge, confidence, empowerment, and resilience. ∙ Higher cost-per-head and lower reach with opportunity for behavioral impact. ∙ Evaluation less complex than tier 3, involving pre- and post-program surveys.
Basic Education	∙ Knowledge and understanding	General diabetes information. Sessions and events for people with, or at risk of, diabetes.	1	∙ Measures of knowledge, awareness, and engagement. ∙ No anticipated behavioral impact. ∙ Includes post-evaluation only.

### Quality standards and assessment

#### Quality standards

Eight quality standards were developed for DSMES (Table 2). It was expected that programs meeting these standards would be more likely to achieve the outcomes and indicators adopted in the National Evaluation Framework compared to programs that do not meet the standards.

**Table 2:** Quality Standards for NDSS Diabetes Self-Management Education and Support

Standard	Description
1	Structured diabetes education should be offered to consumers as soon as possible after diagnosis and on an ongoing basis.
2	Structured diabetes education should be person-centered, use a variety of techniques to promote active learning, and be flexible enough to meet different needs.
3	Structured diabetes education should be provided by an appropriately trained facilitator.
4	Structured diabetes education should be evidence-based, reflect current clinical guidelines, and cover the four key indicators.
5	Structured diabetes education programs should strive to be equitable and accessible to all people.
6	Structured diabetes education should include a written curriculum with clearly defined learning aims and objectives.
7	Structured diabetes education content and resources must be assessed for and meet appropriate readability and health literacy levels.
8	Structured diabetes education should include evaluation that measures program aims and objectives, program fidelity and supports continuous quality improvement.

#### Assessment

The assessment and review of existing programs resulted in the identification of a suite of programs meeting the quality standards. These programs were recommended and subsequently nominated for national delivery by the NDSS administrator, Diabetes Australia. Programs included two comprehensive DSMES - OzDAFNE, for people with type 1 diabetes, and DESMOND for people with type 2 diabetes. Five topic specific programs met the quality standards, including programs related to self-management of carbohydrate intake (CarbSmart), food shopping and interpreting nutrition labels (ShopSmart), glucose monitoring (MonitorSmart), foot care (FootSmart), and managing medication (MedSmart).

Gaps in service provision were then noted, including a lack of programs to support physical activity, self-management of insulin and the use of insulin pumps. To fill this gap, two programs initially assessed as not meeting the standards went through a quality improvement process and were subsequently re-assessed, resulting in inclusion in the national program suite. A new program, ‘Ready Set Go – Let’s Move’ was also developed to support people with diabetes to safely participate in physical activity. All other programs that did not meet the requisite quality standards were no longer supported for delivery through the NDSS.

## Discussion

This National Evaluation Framework has led to nationally consistent delivery of evidence based, person-centered DSMES, including standardized curriculum and facilitator training. The Framework was fully implemented across all NDSS diabetes education and support programs by 2017. Data collection and analysis continue, to facilitate ongoing evaluation and quality improvement.

As health care costs continue to increase, government-funded organizations are under increasing pressure to demonstrate that programs and services are cost-effective, impactful, and achieve targeted outcomes. Effective evaluation of programs and services and transparency in the expenditure of public funds is critical. The National Evaluation Framework helps to maintain accountability in terms of the justification of government spending, and importantly, to people in Australia living with diabetes.

The Framework represents an innovative and comprehensive approach to achieving national consistency in quality and delivery and evaluation of DSMES. In addition to providing a guide to the evaluation of diabetes programs, like that of the CDC’s Framework for Program Evaluation in Public Health adopted in the United States [15, 44] and the DEFINE program in Canada [17], the Australian National Evaluation Framework also provides an exemplar of a consultative approach to the synthesis of a suite of programs delivered by independent service providers across diverse settings. The methods described in this article may guide program administrators and service-providers in other regions around the world, and those providing services for other clinical populations, or other chronic conditions that have a significant requirement for self-management support. Moreover, the Framework could be implemented or adapted for use by other service providers who administer multiple diabetes education programs across a range of settings.

A participatory approach involving service providers, scheme administrators, and evaluation professionals resulted in consensus around the National Evaluation Framework within the NDSS. The Framework provided for the categorization of the diabetes education and support programs available nationally through the NDSS, and guides evaluation processes based on targeted indicators. Moreover, for the first time in Australia, quality standards for diabetes education and support facilitated through the Australian Government-funded NDSS were developed and implemented. These standards ensure that all NDSS programs are of the highest quality, are person-centered, and contain key aspects known to be associated with optimal consumer outcomes, in addition to ensuring that programs comply with the Australian NSQHS.

### Strengths, limitations, and future directions

The approach taken to develop the Framework was collaborative, systematic, and informed by evidence. The establishment and funding of a NET was critical to the development and implementation of the Framework and represents a clear strength of this work. Furthermore, the continued resourcing of a team of specialized evaluation experts ensures ongoing implementation and refinement of the Framework, thereby allowing for continued critical assessment and quality improvement of Australian Government-funded NDSS programs.

The development of the Framework involved extensive consultation with stakeholders to ensure acceptability among NDSS administrators and Agents. The credibility of the Framework and the process was enhanced by the contributions of the ERG who contributed extensive experience in diabetes care. The establishment and implementation of common objectives and quality standards supports nationally consistent delivery of high-quality programs targeting factors known to be associated with improved outcomes among people with diabetes by service providers across all states and territories of Australia. Moreover, the application of standardized evaluation processes utilizing evidence-based instruments for the assessment of outcomes ensures accountability and transparency in service delivery and expenditure of public funds.

Despite the strengths of this work, there are limitations that should be acknowledged. Firstly, greater input from Australians with diabetes could have allowed for the identification of additional factors to be considered when developing the Framework. Secondly, the benefits of rigorous and detailed program evaluation need to be balanced against the availability of resources, accessibility of information, and the need to ensure people who take part in the programs are not overburdened. Based on the available evidence, it is anticipated that the provision of DSMES that improve knowledge and understanding, self-management, self-determination, and psychosocial adjustment will lead to improved clinical outcomes and reduced economic burden at the individual and societal level [20]. However, direct evaluation of these outcomes is not feasible given resources, readily available information, and the interests of NDSS consumers. Moreover, the objectives, quality standards, and measurement instruments were developed or selected based on their suitability to the general population of people with diabetes and may not be suitable to all sub-populations (e.g., culturally and linguistically diverse, Aboriginal and Torres Strait Islanders). The development and refinement of national evaluation tools suitable for application within these populations remains a priority.

## Conclusions

The development of the National Evaluation Framework, including specification of key outcomes, indicators, objectives and tools, and evaluation processes, utilized a systematic and collaborative approach. The development and implementation of quality standards ensured that NDSS programs are nationally consistent, high quality, and target objectives known to be associated with optimal diabetes self-management, improved clinical outcomes, and reduced financial burden. Ongoing assessment of the effectiveness of NDSS programs facilitates the identification of programs achieving desired outcomes and provides opportunities for quality improvement. The data generated through the implementation of the Framework informs service planning and delivery, ensuring optimal impact on the health and well-being of people with diabetes, and a high degree of accountability in the expenditure of public funds.

## Supplementary Information

**Additional file 1.**

## Data Availability

Data sharing is not applicable as no datasets were generated or analyzed.

## Abbreviations

DAFNE: Dose Adjustment for Normal Eating
DESMOND: Diabetes Education and Self-Management for Ongoing and Newly Diagnosed
DES-SF: Diabetes Empowerment Scale - short form
DSMES: Diabetes self-management education and support
ERG: Expert Reference Group
HCCQ: Health Care Climate Questionnaire
NDSS: National Diabetes Services Scheme
NDSS Agents: State and Territory Agents delivering services on behalf of the NDSS
NET: National Evaluation Team
NPS: Net Promotor Score
OzDAFNE: Dose Adjustment for Normal Eating (Australian edition)
PAID: Problem Areas in Diabetes Scale
