# Supplementary figures and images for: The design of an evaluation framework for diabetes self-management education and support programs delivered nationally
Source: BMC Health Serv Res. 2022 Jan 9;22:46. doi: 10.1186/s12913-021-07374-4 (PMC8744356; doi:10.1186/s12913-021-07374-4)

**Appendix A**

**
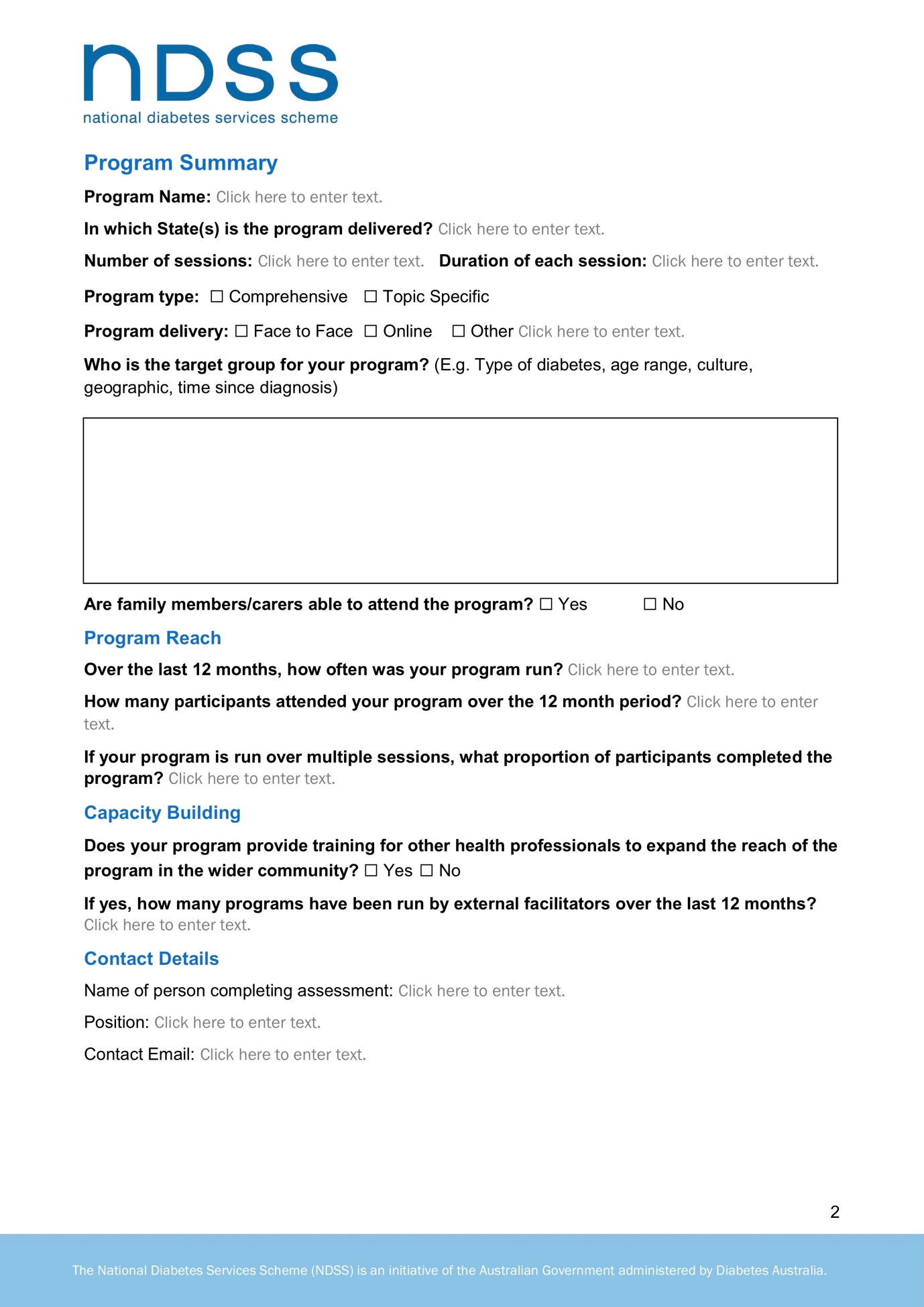
**

**
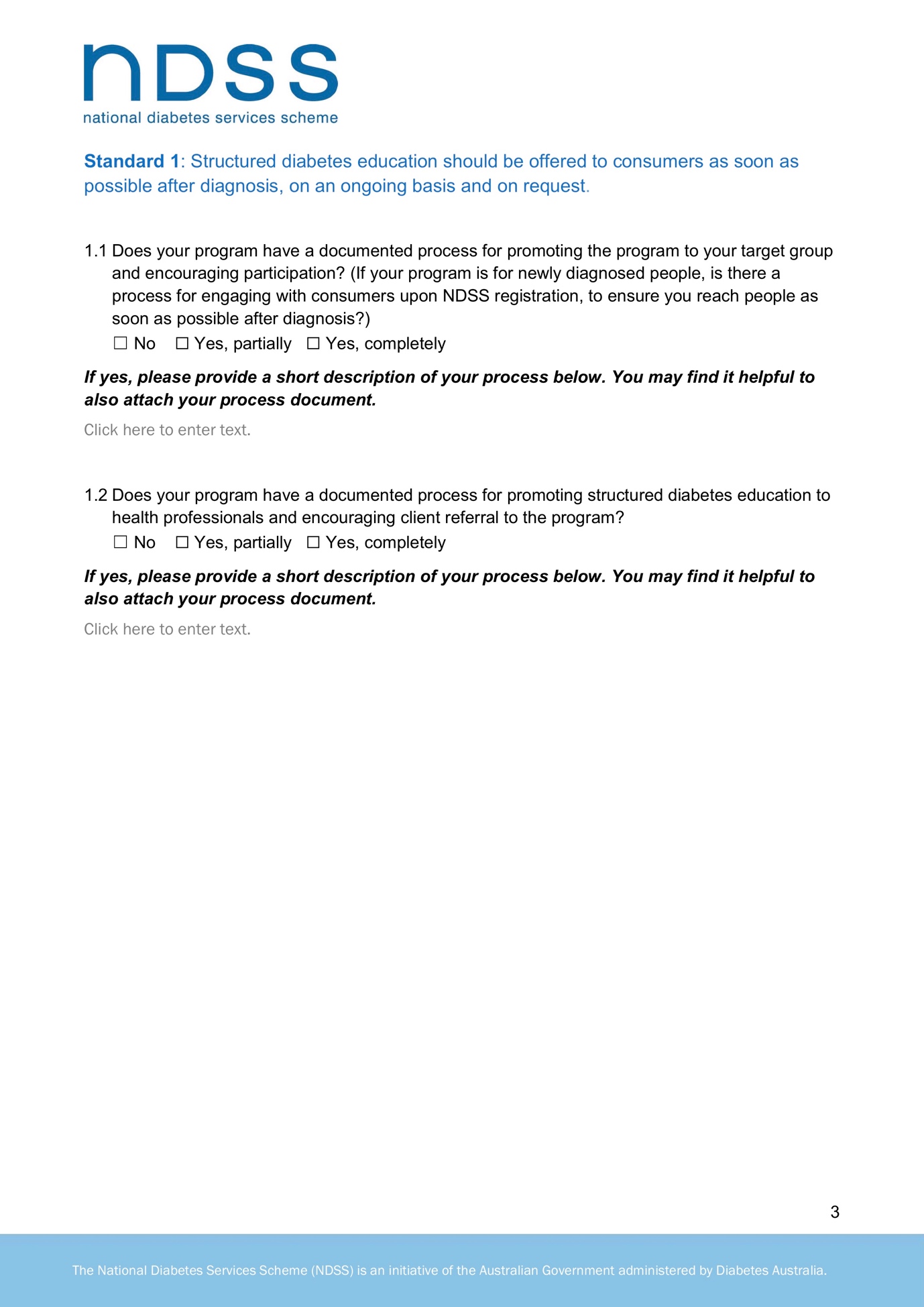
**

**
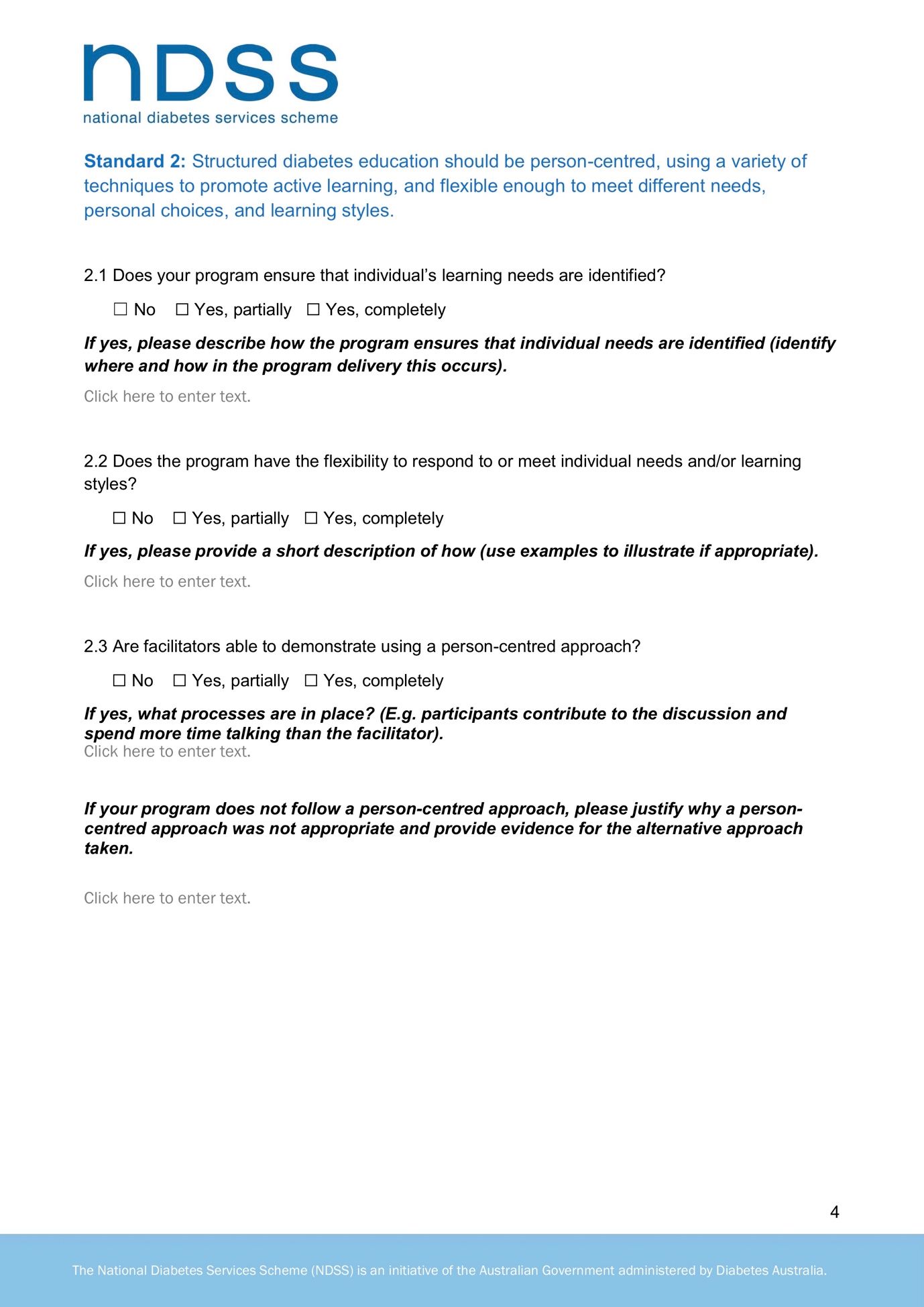

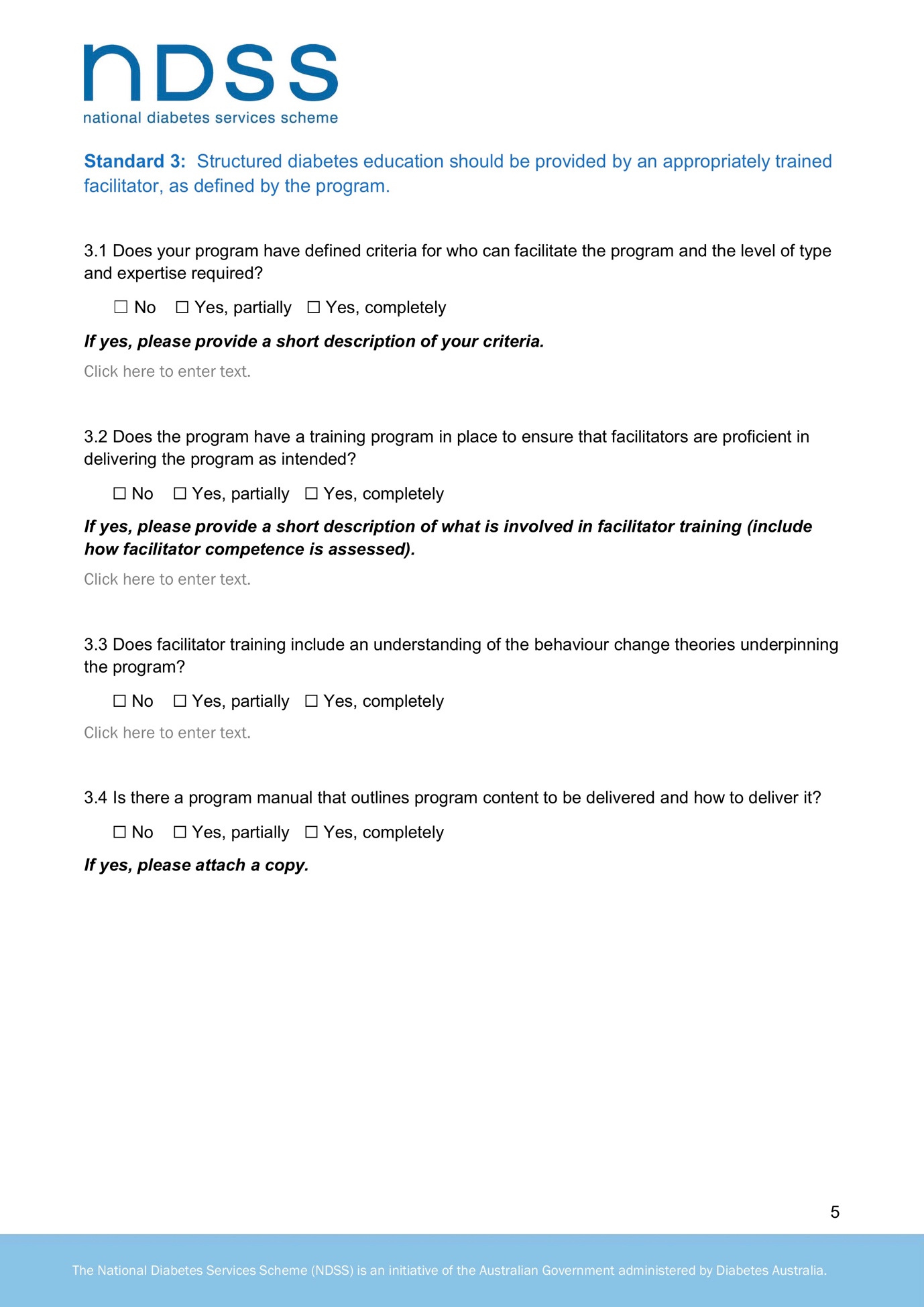
**

**
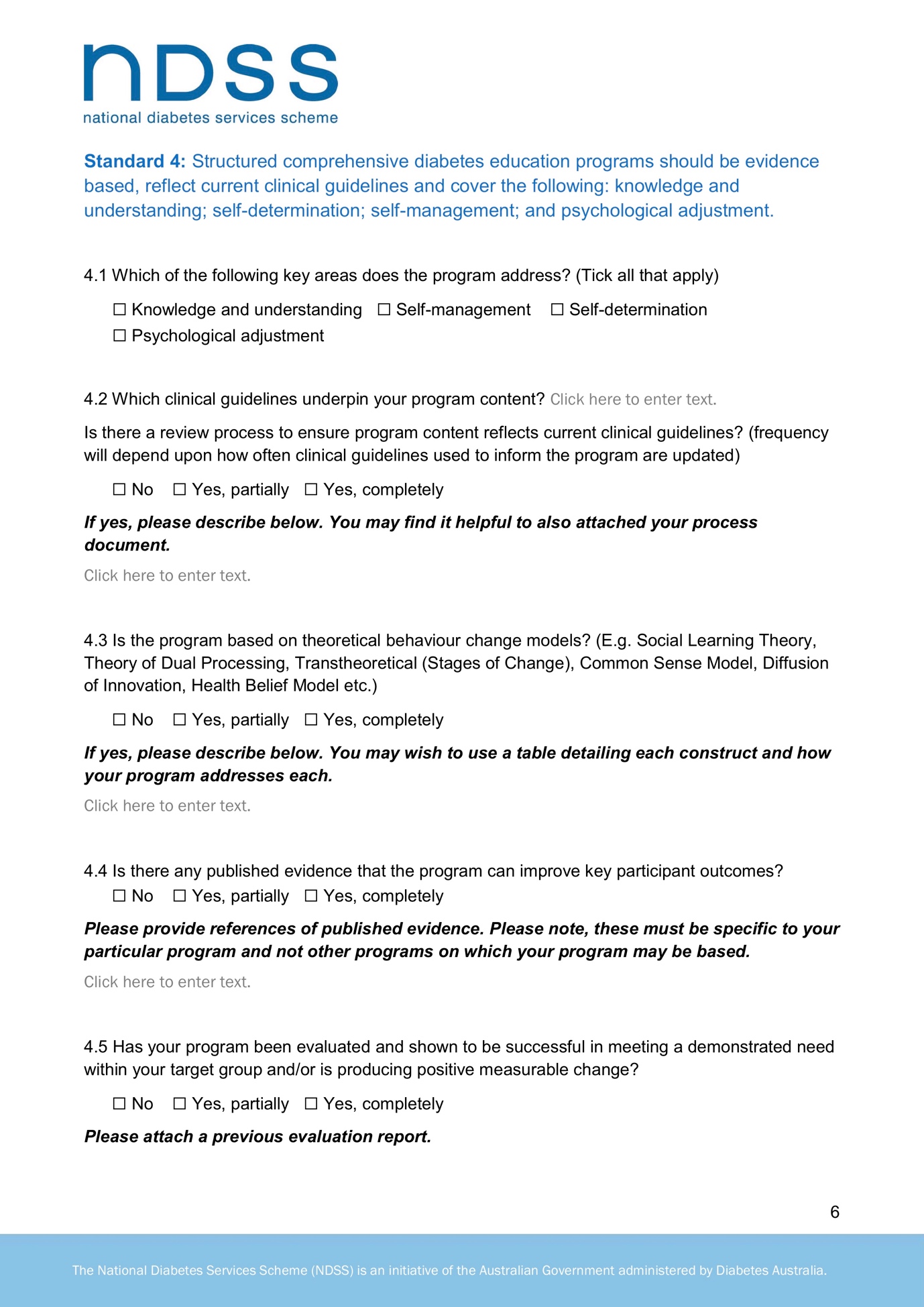
**

**
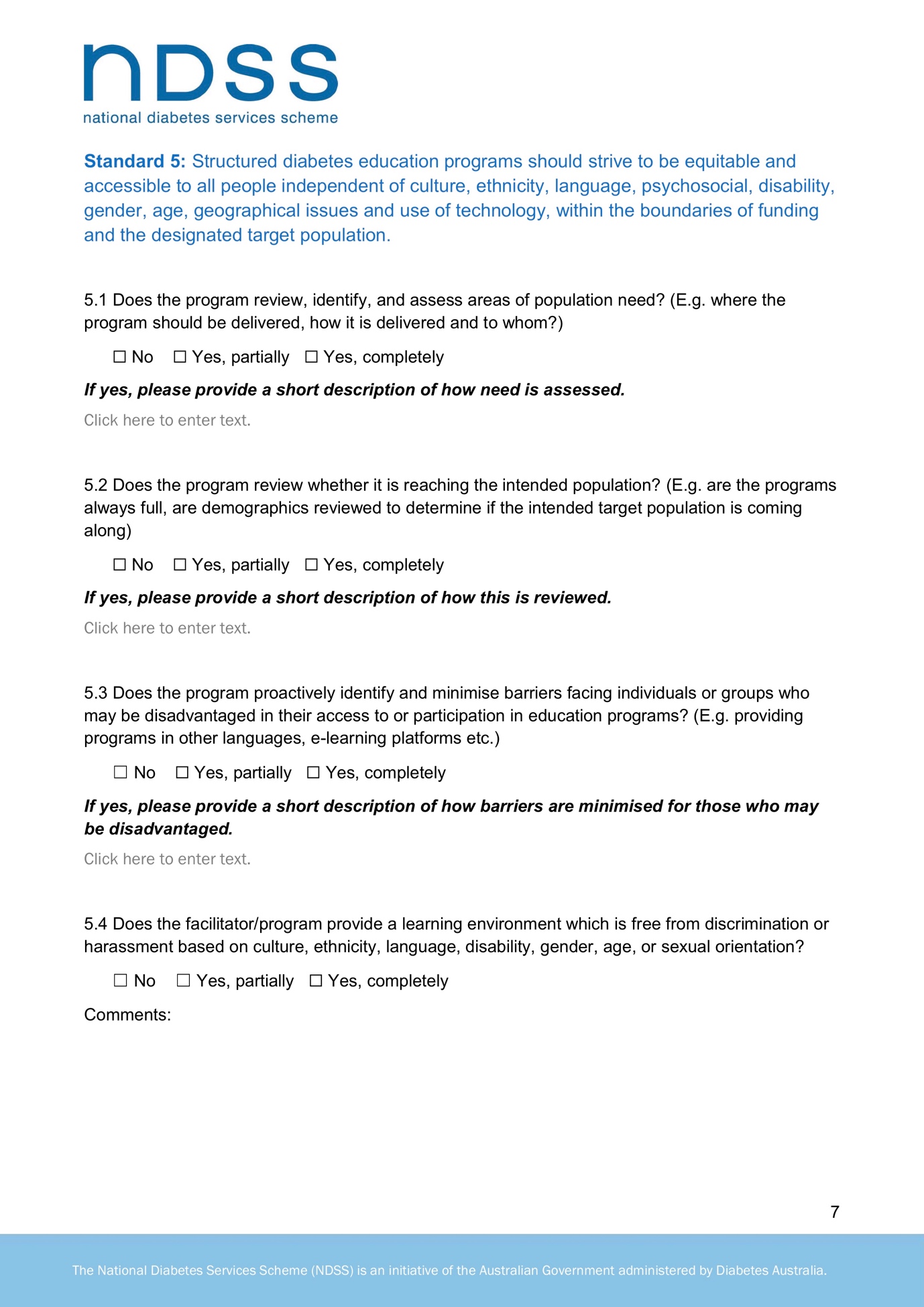
**

**
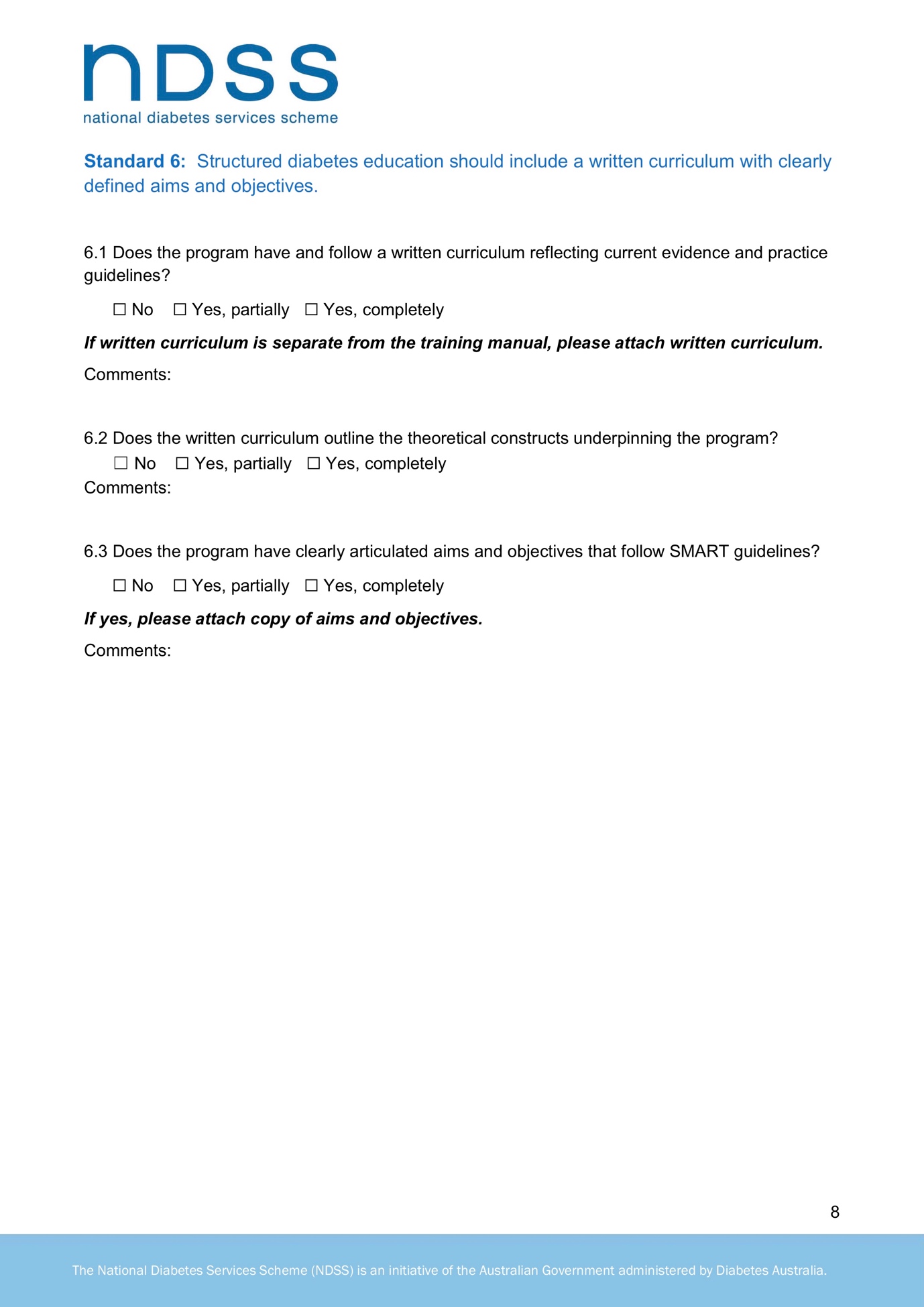
**

**
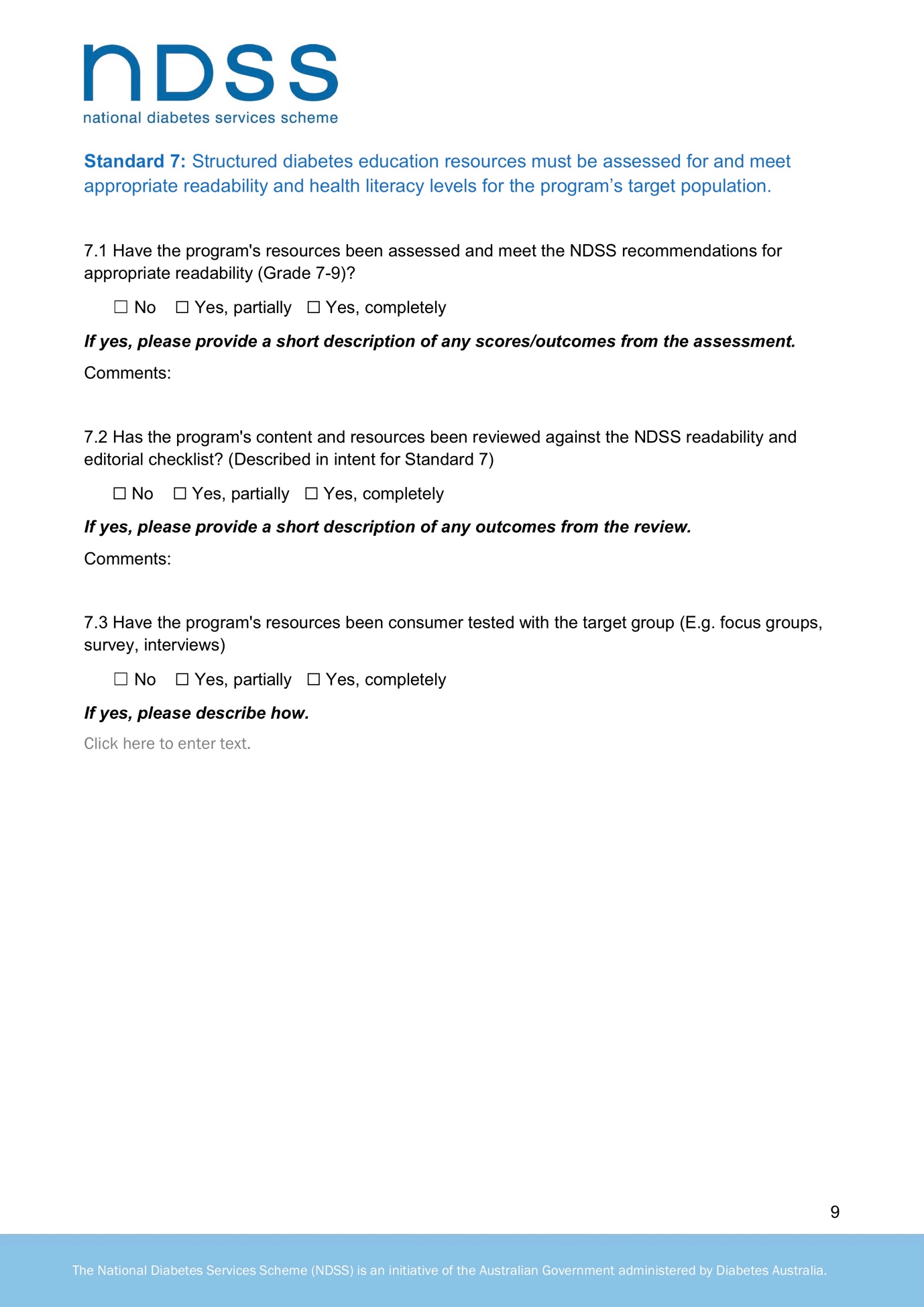
**

**
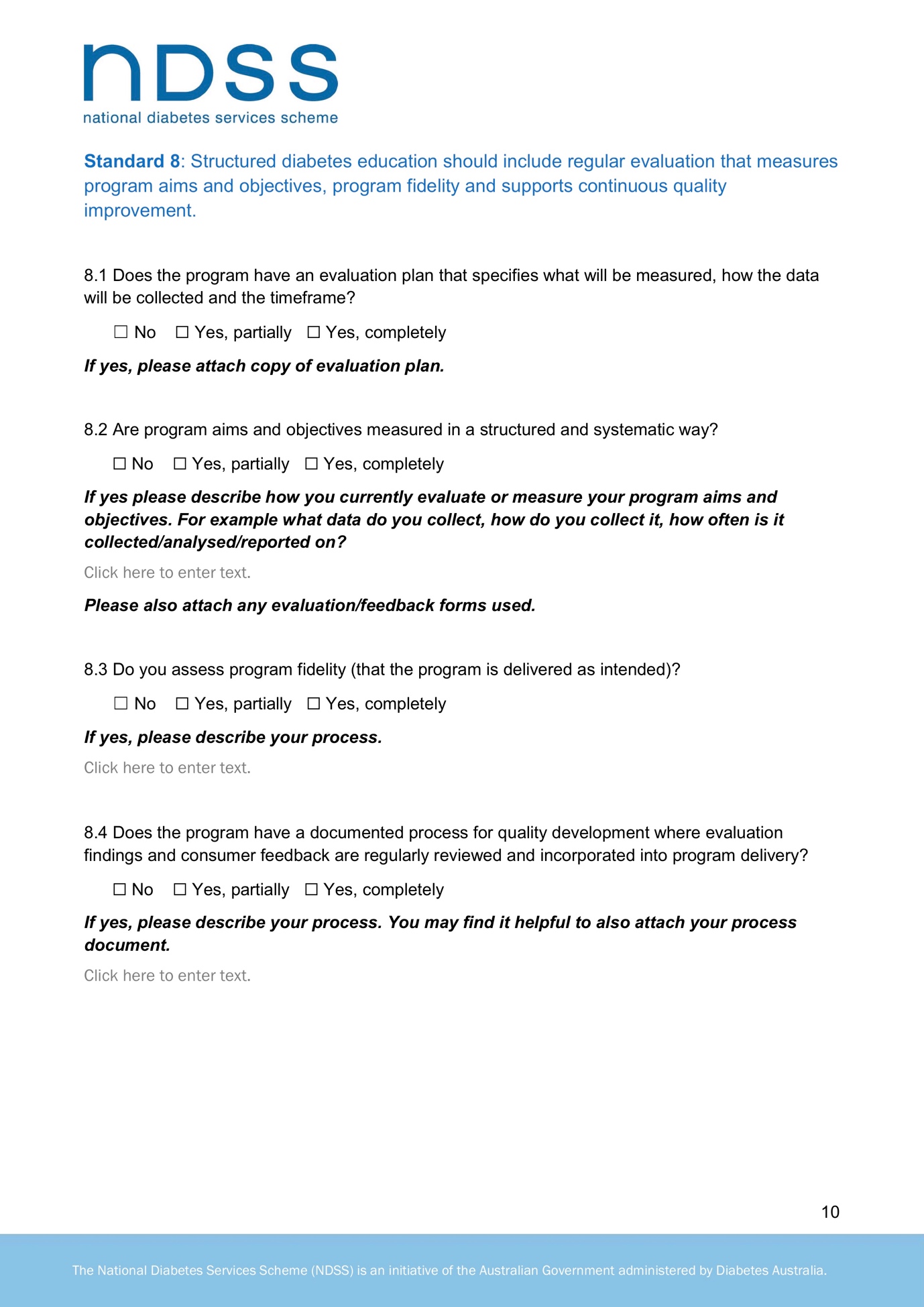
**

**
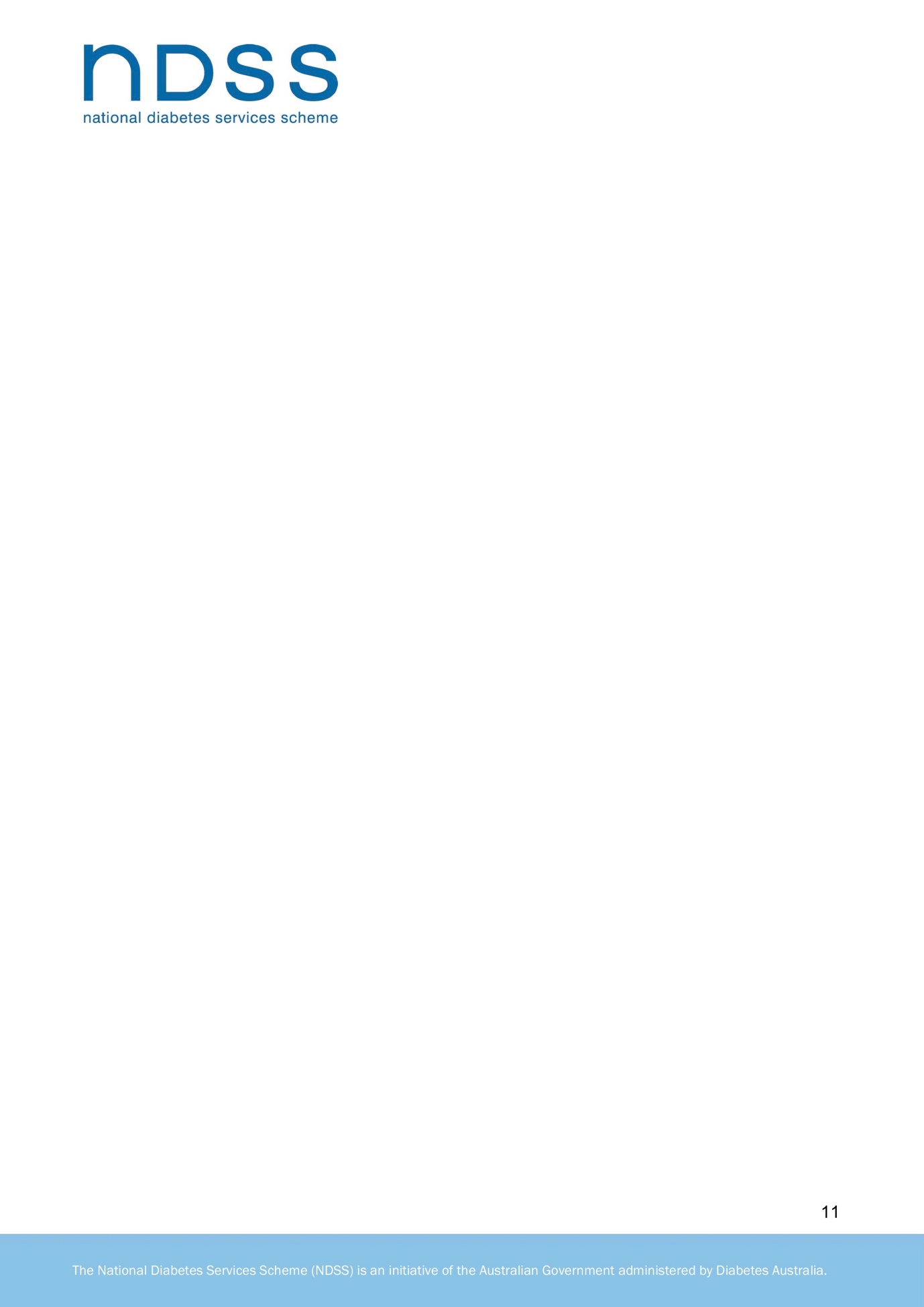
**

**
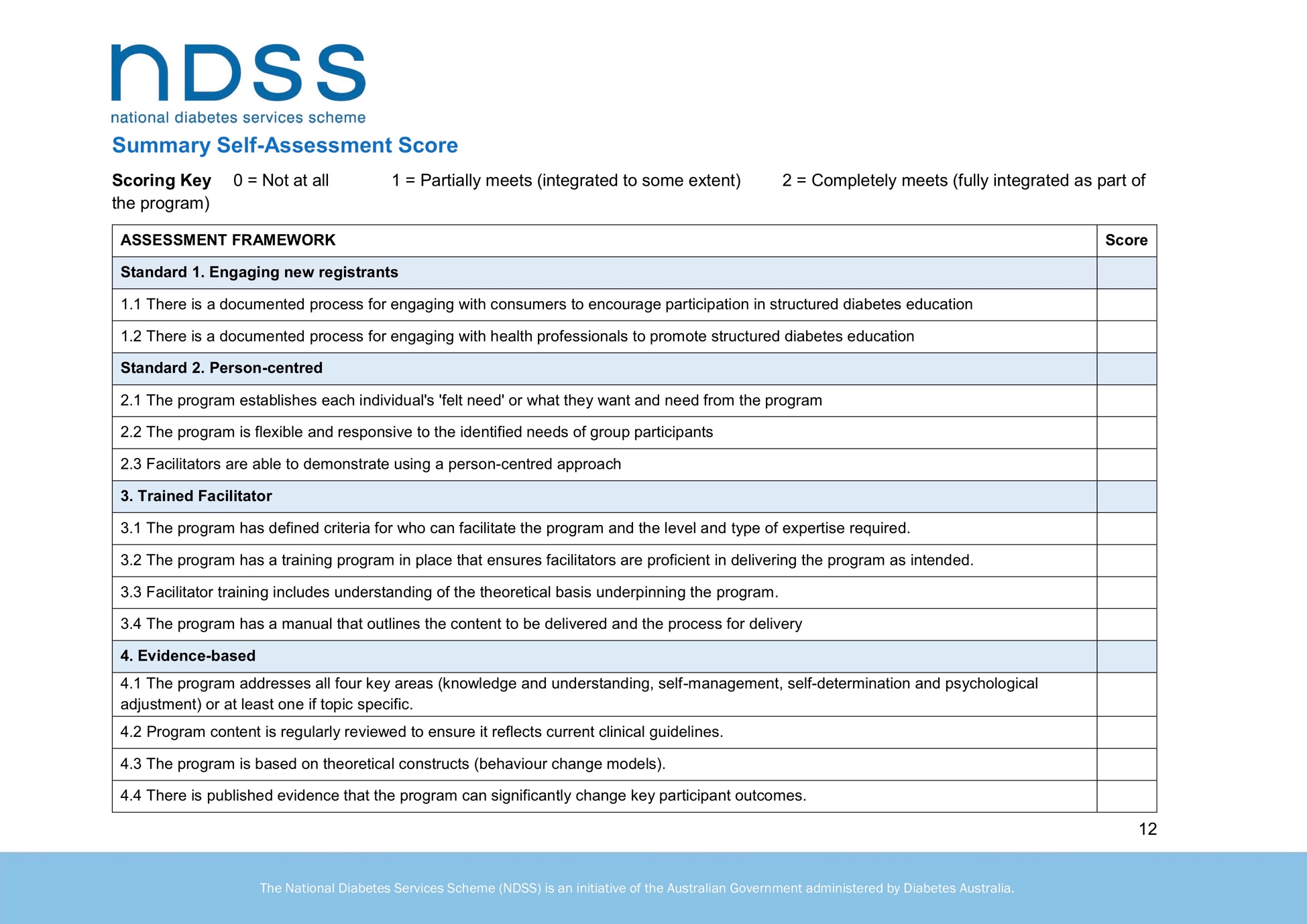
**

**
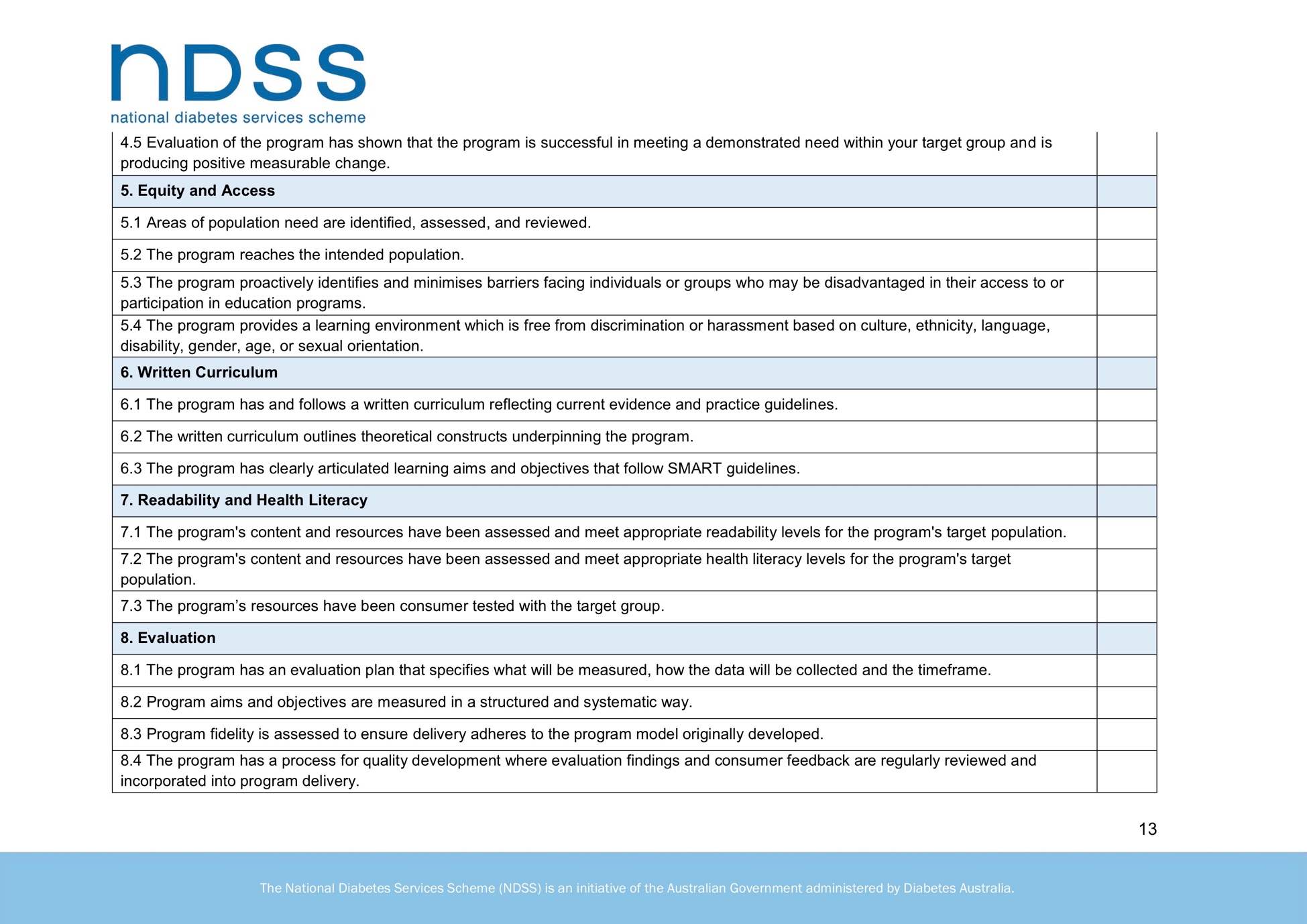
**

**
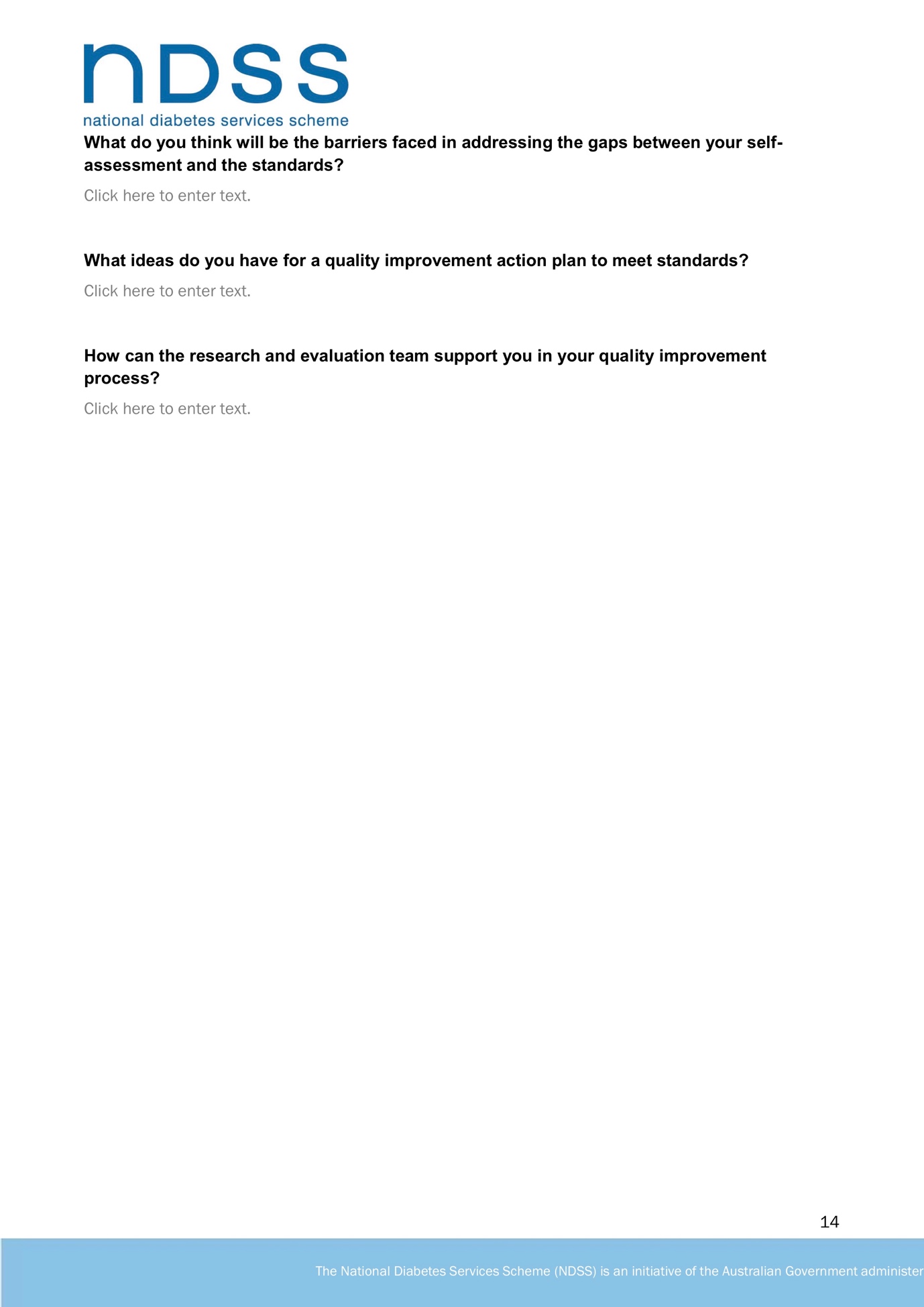
**

Supplement: Supplementary file 1 — Additional file 1. [file 12913_2021_7374_MOESM1_ESM.docx]
